# *FCGR2A* Gene Polymorphism Association in Children with Multisystem Inflammatory Syndrome

**DOI:** 10.1007/s13312-025-00047-z

**Published:** 2025-04-11

**Authors:** Esra Yeşiltepe, Derya Duman, Necdet Kuyucu, Sevcan Tuğ Bozdoğan, Lara Çıtırık, Edanur Yeşil, Derya Karpuz

**Affiliations:** 1 Department of Pediatrics, Faculty of Medicine, Mersin University, 33343 Mersin, Turkey; 2 Department of Pediatric Cardiology, Faculty of Medicine, University of Mersin, 34. Cadde, Ciftlikkoy Kampusu, 33343 Mersin, Turkey; 3 Department of Pediatric Infectious Disease, Faculty of Medicine, University of Mersin, 34. Cadde, Ciftlikkoy Kampusu, 33343 Mersin, Turkey; 4 Department of Medical Genetics, Faculty of Medicine, Çukurova University, Balcalı Kampüsü, 01330 Adana, Turkey; 5 Department of Pediatric Cardiology, Faculty of Medicine, Mersin University, 33343 Mersin, Turkey

**Keywords:** Cardiovascular system, Child, Genetics, Multisystem inflammation

## Abstract

**Objective:**

Fc gamma receptor IIa (*FCGR2A*) gene polymorphism is associated with increased susceptibility to autoimmune and infectious diseases. The aim of the present study was to evaluate the association of *FCGR2A* rs1801274 polymorphism with the development and severity of multisystem inflammatory syndrome in children (MIS-C).

**Methods:**

This case-control study was conducted in a single center with MIS-C patients and healthy children. Clinical and cardiac imaging data of the participants was collected. The association between the clinical severity of the disease and *FCGR2A* rs1801274 polymorphism were investigated.

**Results:**

There was no significant association between *FCGR2A* rs1801274 polymorphism and cardiovascular complications in MIS-C patients. However, those with homozygous *FCGR2A* rs1801274 gene polymorphism developed severe cardiac dysfunction and required immunomodulatory agents other than intravenous immunoglobulin. The mean age of the patients with severe MIS-C was significantly higher than those with mild MIS-C, and systolic dysfunction was significant.

**Conclusions:**

Further multicenter studies in different ethnic groups are needed to evaluate the association between differences in the *FCGR2A* rs1801274 gene and severity of MIS-C and/or other inflammatory diseases.

**Trial Registry:**

Mersin University Clinical Trial Registry, Decision number 2022/280 dated April 20, 2022.

## Introduction

Multisystem inflammatory syndrome in children (MIS-C) is a novel syndrome identified in the global coronavirus disease 2019 (COVID-19) pandemic, seen in the pediatric population exposed to severe acute respiratory syndrome coronavirus 2 (SARS-CoV-2) [1–3]. Results thus far suggest that MIS-C is an immunologically mediated disease associated with previous SARS-CoV-2 infection [4, 5]. Although the clinical features of this syndrome resemble Kawasaki disease (KD), MIS-C is a disease with multiple organ damage, especially cardiac involvement, and differs from KD in terms of the degree of hyperinflammation and dysregulated immune responses [1, 3, 5].

Fc gamma receptor IIa (*FCGR2A*) gene encodes the FcγRIIA protein, which belongs to a family of immunoglobulin G receptors found on phagocytic cells such as natural killer cells, macrophages and neutrophils and is involved in the phagocytic removal of antigen–antibody complexes [6, 7]. Several *FCGR* gene variants have been associated with increased susceptibility to autoimmune diseases and variable response to infections [8, 9]. MIS-C is a KD-like disease triggered by viral infection and characterized by hyperinflammation [7]. Studies have associated the single nucleotide polymorphism (SNP) rs1801274 in *FCGR2A* with increased susceptibility to KD [10]. Specific variants of FcγR have been reported to be associated with disease severity and response to intravenous immunoglobulin (IVIg) in KD [6, 11, 12]. MIS-C has multiorgan involvement due to a severe inflammatory response and requires intensive care unit admission more frequently than KD. Recent studies have suggested that gene polymorphisms caused by certain mutations may result in an excessive inflammatory response and may be associated with the development of MIS-C [13, 14]. In this study, the association between *FCGR2A* rs1801274 polymorphism and the risk of developing MIS-C and disease severity were evaluated.

## Methods

This single-center, case control study was conducted between October 2020 and April 2022 in the Departments of Pediatric Cardiology and Pediatric Infection of a University Hospital in Turkey. Informed consent was obtained from participants prior to participation in the study. The study was approved by the institutional ethics committee prior to the study.

Patients diagnosed with MIS-C according to Centers for Disease Control and Prevention and World Health Organization criteria [3, 15], and age- and sex-matched healthy children were included in the study. Based on severity, patients with MIS-C were categorized as mild or severe [16]. The heart rate, blood pressure, echocardiographic (ECHO) and electrocardiographic (ECG) parameters of all of the children were recorded. The biochemical data [leukocyte count, hemoglobin, serum aspartate aminotransferase, serum alanine aminotransferase, serum albumin, and C-reactive protein (CRP), serum procalcitonin (PCT), erythrocyte sedimentation rate, D-dimer, and cardiac biomarkers like N-terminal pro-brain-natriuretic peptide (NT-proBNP) and troponin I, serum creatine kinase (CK), serum creatine kinase-myocardial band (CK-MB)] were assessed in the patient group.

Transthoracic echocardiography (Vivid E9 Pro Ultrasound System, GE Healthcare Canada, Inc., Toronto, ON, Canada) was performed using 3 and 6 MHz transducers and by 2D, M-mode and colored Doppler, conventional continuous-wave (CW), and pulse wave (PW) Doppler visualization methods. Left ventricular (LV) structure and systolic and diastolic functions were measured according to the pediatric guidelines of the American Society of Echocardiography [17]. Cardiotoxicity was defined as abnormally low LV systolic function—LV fractional shortening (FS) < 28% and LV ejection fraction (EF) < 57%. Diastolic dysfunction was defined as an early filling velocity (E)/late filling velocity (A) ratio (E/A) greater than 2 or less than 1.

Genomic DNA (gDNA) was isolated from the peripheral blood samples using the QIAsymphony DSP DNA Mini Kit (Qiagen GmbH, Hilden, Germany) with an automated QIAsymphony DNA isolation device (Qiagen GmbH). DNA concentration measurements were performed via a Qubit 4.0 Fluorometer (Thermo Fisher Scientific Inc., Waltham, MA, USA) for quality control after isolation.

The *FCGR2A* gene p.H131R (rs1801274) polymorphism was analyzed using real-time Polymerase Chain Reaction (PCR) (Bio-Rad CFX96, Hercules, CA, USA), with HOT FIREPol Probe qPCR Mix Plus (ROX) 5x (Solis BioDyne OÜ, Tartu, Estonia), TaqMan Custom SNP genotyping assays (40X and 80X), and TaqMan DME genotyping assays (20X) (Thermo Fisher Scientific Inc.). PCR program included 40 cycles: 20 s at 95 °C for denaturation and 1 min at 60 °C for primer binding and elongation. Following the completion of the PCR process, the results were analyzed for both alleles.

*Statistical analysis:* Data were analyzed using SPSS Statistics for Windows version 23.0 (IBM Corp., Armonk, NY, USA). Kolmogorov–Smirnov and Shapiro–Wilk tests were administered to assess the normality of numerical data. Descriptive statistics were employed for both the continuous and discrete digital variables, as median (IQR) and numbers (percentage), respectively. Categorical variables across distinct groups were compared using the Pearson Chi-square test and the Fisher’s Exact test. Numerical data between two groups were compared using the Mann–Whitney U Test. *P* < 0.05 was accepted as statistically significant.

## Results

A total of 35 patients [12 girls, median (IQR) age 10 (6,12) years] and 36 age- and sex-matched healthy controls (13 girls, median (IQR) age 12 (7, 14) years] were included. All patients with MIS-C had KD phenotype. There were 14 and 21 cases of mild and severe MIS-C, respectively. Children with severe MIS-C were significantly older compared to those with mild MIS-C [median (IQR) age 11 (6.5, 14) years vs 6 (5, 11.2) years; *P* = 0.028]. The median systolic and diastolic blood pressure values were significantly lower in MIS-C patients compared to the controls (*P* = 0.001 and *P* = 0.021, respectively). Cardiac and inflammatory markers were significantly higher in the patients with MIS-C compared to controls (*P* < 0.01). Although the median NT-proBNP values were higher in severe MIS-C patients, the difference was not statistically significant (*P* = 0.235).

The LV EF and FS median values were lower in the patients with severe MIS-C compared to the mild MIS-C patients (*P* = 0.001 and *P* = 0.003, respectively). In the MIS-C patients, the median heart rate was significantly higher, and the median QT dispersion (QTd) values were lower compared to the controls (*P* < 0.001 and *P* = 0.014, respectively). The median duration of hospitalization was significantly longer in the severe MIS-C patients than in the mild MIS-C patients (*P* = 0.006). The demographic, ECHO, ECG and laboratory characteristics of all of the groups are shown in Table 1. Conventional M-mode echocardiography results of the patient groups are shown in Table 2.

**Table 1:** Demographic, clinical and laboratory profile of the study participants

	Severity of disease		*P value*	Study Group		*P value*
	Mild MIS-C (*n* = 14)	Severe MIS-C (*n* = 21)		MIS-C (*n* = 35)	Control (n = 36)	
*Demographic and Clinical*						
Age (year)^*a*^	6 (5–11.2)	11 (6.5–14)	0.028^*c*^	10 (6–12)	12 (7–14)	0.119^*c*^
*Gender*^*b*^						
Female	5 (35.7)	7 (33.3)	0.999^*d*^	12 (34.3)	13 (36.1)	0.872^*d*^
Male	9 (64.3)	14 (66.7)		23 (65.7)	23 (63.9)	
Systolic BP (mmHg)^*a*^	110 (98.0–111.3)	100 (87.5–111)	0.263^*c*^	107 (96–111)	115 (107.8–120)	0.001^*c*^
Diastolic BP (mmHg)^*a*^	70 (63.3–75.0)	65 (51–71.5)	0.309^*c*^	68 (58–72)	72 (62.8–78)	0.021^*c*^
Duration of hospital stay (days)^*a*^	7.0 (4.8–8.0)	8.0 (7.0–14.0)	0.006^*c*^	8 (7–11)	–	
*Cardiovascular system pathology*^*b*^						
No	7 (50)	6 (28.6)	0.199^*d*^	13 (37.1)	36 (100)	< 0.001^*d*^
Yes	7 (50)	12 (71.4)		22 (62.9)	0	
*Laboratory*						
Hb (g/dL)^*a*^	10.8 (9.9–11.4)	11.5 (10.3–12)	0.154^*c*^	11.2 (10.2–11.8)	12.3 (11–14.0)	0.001^*c*^
HCT (%)^*a*^	31 (29–32.5)	33 (30–34)	0.222^*c*^	31.0 (29.0–34.0)	36 (33–41.5)	< 0.001^*c*^
Troponin I (ng/mL)^*a*^	21.5 (3.5–70.8)	19 (3–32)	0.654^*c*^	19.0 (3.0–47.0)	2.5 (1–8.3)	< 0.001^*c*^
ProBNP (pg/mL)^*a*^	1274 (156–11,148.3)	2934 (305.5–20,326)	0.235^*c*^	1790 (197–15,439)	107.5 (71–178.5)	< 0.001^*c*^
D-dimer (mg/L)^*a*^	2.3 (1.1–3.1)	3.2 (2–4.8)	0.083^*c*^			

Values expressed as ^a^median (IQR), ^b^n (%)

BP Blood pressure, Hb Hemoglobin, HCT Hematocrit, IQR Interquartile range, MIS-C Multisystem inflammatory syndrome in children, ProBNP Pro B-type natriuretic peptide

^c^Mann-Whitney U Test

^d^Pearson Chi square Test

**Table 2:** Electrocardiographic and echocardiographic measurements of the groups

	Severity of MIS-C		*P value*	Study Group		*P value*
	Mild (n = 14)	Severe (n = 21)		MIS-C (n = 35)	Control (n = 36)	
*Electrocardiographic parameters*						
Heart rate (bpm)	97.5 (79.0–125.0)	100.0 (82.0–135.0)	0.855^a^	100.0 (81.0–135.0)	83.0 (68.8–92.5)	< 0.001^a^
PR interval (msec)	110.0 (96.3–125.0)	120.0 (96.0–139.0)	0.516^a^	110.0 (99.0–138.0)	120.0 (100.0–127.5)	0.342^a^
QRS duration (msec)	80.0 (78.0–100.0)	80.0 (80.0–91.5)	0.987^a^	80.0 (80.0–96.0)	82.5 (80.0–100.0)	0.184^a^
QT interval (msec)	350.0 (337.0–361.5)	350.0 (345.0–370.0)	0.434^a^	350.0 (340.0–367.0)	350.0 (350.0–387.5)	0.282^a^
QTc (msec)	410.0 (400.0–442.5)	420.0 (390.0–445.0)	0.960^a^	420.0 (400.0–440.0)	405.0 (390.0–430.0)	0.143^a^
QTd (msec)	93.5 (44.3–118.8)	58.0 (45.0–115.0)	0.677^a^	69.0 (47.5–110.0)	130.0 (105.0–140.0)	0.014^a^
*Echocardiographic parameters*						
LVEDD (mm)	37.0 (29.8–42.3)	39.0 (33.0–42.0)	0.654^a^	44.5 (38.3–46.8)	39.0 (32.0–42.0)	0.003^a^
EF (%)	71.0 (66.8–75.3)	65.0 (56.0–67.5)	0.001^a^	67.0 (62.0–71.0)	67.5 (65.0–73.0)	0.461^a^
FS (%)	38.0 (35.8–44.3)	35.0 (29.0–37.0)	0.003^a^	36.0 (33.0–38.0)	38.0 (35.3–41.8)	0.144^a^
Mitral E velocity (cm/sec)	0.97 (0.87–1.00)	0.90 (0.84–0.96)	0.118^a^	0.92 (0.86–0.98)	0.99 (0.92–1.05)	0.010^a^
Mitral A velocity (cm/sec)	0.54 (0.48–0.60)	0.48 (0.45–0.54)	0.175^a^	0.50 (0.46–0.56)	0.56 (0.50–0.67)	0.015^a^
E/A rate	1.82 (1.52–1.90)	1.88 (1.46–2.04)	0.654^a^	1.84 (1.51–1.96)	1.71 (1.48–1.88)	0.157^a^

Values expressed as median (IQR)

EF Ejection fraction, FS Fractional shortening, LVEDD Left ventricle end diastolic dDiameter, MIS-C Multisystem Inflammatory Syndrome in Children, QTc Corrected QT interval, QTd QT dispersion

^a^Mann-Whitney U Test

NA was extracted and genotyped for *FCGR2A r*s1801274; c.G497A from 35 patients and 36 controls. The frequencies of the genotypes are summarized in Table 2. There was no statistically significant difference in terms of the *FCGR2A* rs1801274 gene polymorphism in various MISC subtypes nor did it differ from control samples. *FCGR2A* rs1801274 AA genotype was more common in the severe MIS-C patients (45.7%) (Table 3). Of these severe patients, one patient underwent stenting for right coronary artery thrombosis and myocardial infarction, and four developed severe systolic dysfunction, with an EF < 55%. Of the patients with severe systolic dysfunction, two required immunomodulatory therapy (interleukin-1 receptor antagonist) in addition to standard treatment for MIS-C, and one patient had ventricular tachycardia. The alleles of the analyzed SNP gene did not correlate with the ECHO, ECG, and laboratory parameters.

**Table 3:** Genotype frequencies of the groups

	Severity of MIS-C		*P value*	Study Group		*P value*
	Mild (*n* = 14)	Severe (*n* = 21)		MIS-C (*n* = 35)	Control (*n* = 36)	
*FCGR2A rs1801274 (A > G)*						
AA	4 (28.6)	4 (19.0)	0.615^a^	8 (22.9)	7 (19.4)	0.099^b^
AG	3 (21.4)	8 (38.1)		11 (31.4)	20 (55.6)	
GG	7 (50.0)	9 (42.9)		16 (45.7)	9 (25.0)	

Values expressed as n (%)

MIS-C Multisystem inflammatory syndrome in children

^a^Fisher’s Exact Test

^b^Pearson Chi-square Test

## Discussion

This study is the first to evaluate the association of *FCGR2A* rs1801274 polymorphism with the risk of developing MIS-C and the severity of the disease in children. In the present study, no association was found between the AA genotype of *FCGRA2* rs1801274 and MIS-C in children of Turkish origin. However, *FCGR2A* rs1801274 AA genotype was more frequent in the severe MIS-C patients, at a rate of 45.7%. Furthermore, there was no significant association between this polymorphism and disease severity and cardiovascular biomarkers. When the patients with the *FCGR2A* rs1801274 AA genotype were analyzed individually, it was observed that their clinical course was more severe.

MIS-C is a disease characterized by hyperinflammation that occurs weeks after COVID-19 infection. Although there have been many reports on this subject in the last three years, the pathogenesis of the disease and its triggers have not been clearly elucidated. Although, the clinical features are similar to KD, there are differences in the severity of cardiac involvement, platelet count, D-dimer, and NT-ProBNP levels. Similar to KD, MIS-C is thought to occur as a result of an excessive cytokine response to SARS-CoV-2 virus as a result of a disruption in immune system regulation and genetic predisposition is thought to play an important role in this pathophysiological process [5].

Several gene polymorphisms predispose to the development of MIS-C [18, 19]. Studies have also investigated whether some genes may be associated with the severity of COVID-19 infection. One study showed that the rs1801274 polymorphism in *FCGR2A* is associated with COVID-19 infection severity [18]. This study was planned considering that gene polymorphisms associated with KD may be important in MIS-C due to the similarities in innate immune response in the pathogenesis of KD and MIS-C.

In the present study, no association was found between the AA genotype of *FCGRA2* rs1801274 and MIS-C development and severity in children of Turkish origin, possibly due to ethnic differences and due to a small sample size. Genetic studies on KD have shown that disease prevalence and genetic susceptibility may vary from population to population [20–22]. Although there was no significant difference, when the patients with *FCGR2A* rs1801274 AA genotype were analyzed individually, it was observed that these patients had a more severe clinical course. A patient with a dominant *FCGR2A* rs1801274 allele underwent stenting for right coronary artery thrombosis and myocardial infarction and four patients with dominant polymorphism had an EF < 55% and developed severe systolic dysfunction during follow-up. Of these patients, two required interleukin-1 receptor antagonist treatment in addition to standard MIS-C treatment. One patient had ventricular tachycardia. *FCGR2A* rs1801274 AA genotype was more frequent in the severe MIS-C patients, at a rate of 45.7%, thus predisposing to a longer hospital stay.

The etiopathogenesis of KD is not fully understood but it is most often characterized by hypercytokinemia. There are overlapping signs, symptoms, and other clinical and laboratory findings between Kawasaki and MIS-C of which both are hyperinflammatory diseases. Polymorphisms may have several phenotypic effects associated with the diseases. All gene polymorphisms are not compatible with the studies, and also there are some factors which we cannot change. Challenges like modifiable exposures, as well as the probability of linkage disequilibrium in the genes and polymorphisms of interest exist to identify suitable polymorphisms for studying [23]. There are at least six or more known polymorphisms on *FCGR2* gene but due to our limited resources, we preferred to study the most potential and efficient one [23–25]. In this study, a topic with limited information within a single center’s utilities was conducted and this could also contribute to a different view of this area. This is the first study to compare this gene polymorphism between these two similar diseases in a specific geographic region. The most important limitation of the present study was that it was conducted with a limited number of patients in a single center. The feasibility of performing genetic studies and the rarity of the disease were other limiting factors.

Although there is no MISC epidemic at the moment, it is still important to study the gene polymorphism in inflammatory diseases including MIS-C and Kawasaki disease. Gene polymorphisms examined in a certain geography, race, and population will provide evidence for associations and open avenues for future research.

This study highlights that genetic make-up could lead to variability in the clinical presentation following exposure to COVID-19 virus, and it could help physicians to be aware of the increased risk of cardiovascular events due to genetic variability.

## What this Study adds?

- No significant association was observed between *FCGR2A* rs1801274 polymorphism and cardiovascular complications in MIS-C patients.
- Children with MIS-C with homozygous *FCGR2A* rs1801274 gene polymorphism developed severe cardiac dysfunction

## Data Availability

The data that support the findings of this study are available from the corresponding author upon reasonable request.
